# MED19 Knockdown Induces G1 Cell-Cycle Arrest Through Downregulation of AREG and CKS1B in Lung and Breast Cancer Cells

**DOI:** 10.3390/ijms272110004

**Published:** 2026-11-08

**Authors:** Sailakshmi Iyer, Naoko Hattori, Takeya Nakagawa, Satoshi Fujii, Tomonori Hayashi, Takashi Ito

**Affiliations:** 1 Department of Biochemistry, Nagasaki University Graduate School of Biomedical Sciences, Nagasaki 852-8523, Japan; bb55322805@ms.nagasaki-u.ac.jp (S.I.);; 2 Department of Dermatology, Nagasaki University Graduate School of Biomedical Sciences, Nagasaki 852-8523, Japan; 3 Department of Bioscience and Bioinformatics, Kyushu Institute of Technology, Fukuoka 804-8550, Japan; 4 Radiation Effects Research Foundation, Hiroshima 732-0815, Japan

**Keywords:** *MED19*, AREG, *CKS1B*, G1 arrest

## Abstract

Mediator complex subunit 19 (*MED19*), a component of the Mediator complex, has emerged as a potential regulator of cancer progression, although its downstream transcriptional programs remain poorly understood. Here, we investigated the role of *MED19* across 20 human cancer cell lines. An siRNA-mediated knockdown (KD) screen identified A549 lung cancer and MCF-7 breast cancer cells as particularly sensitive to *MED19* KD, exhibiting markedly reduced cell viability. Flow cytometric analysis revealed that *MED19* KD induced G1-phase arrest, suggesting a role in G1/S-phase progression. RNA sequencing followed by differential expression, Gene Ontology (GO), and chromosomal mapping analyses identified amphiregulin (*AREG*) and cyclin-dependent kinase regulatory subunit 1B (*CKS1B*) as candidate downstream effectors in A549 and MCF-7 cells, respectively. Knockdown of *AREG* or *CKS1B* inhibited cell proliferation and recapitulated the G1-phase arrest induced by *MED19* KD. Moreover, ectopic expression of *AREG* or *CKS1B* partially rescued the cell-cycle defects caused by *MED19* KD. These findings identify *MED19* as a context-dependent regulator of cancer cell proliferation and G1/S-phase progression through distinct downstream transcriptional programs in lung and breast cancer cells.

## 1. Introduction

Mediator is a central component of the eukaryotic transcriptional machinery that interacts with RNA Polymerase II (RNA Pol II) and functions as a molecular bridge between transcription factors (TFs), co-activators, and chromatin-modifying proteins, thereby facilitating pre-initiation complex (PIC) assembly and regulating gene expression [1,2,3]. Although the Mediator complex is evolutionarily conserved from yeast to humans, its composition and structural complexity have significantly expanded during evolution. In Saccharomyces cerevisiae, the Mediator complex consists of 21 subunits with a molecular weight of approximately 0.8 MDa, whereas the human Mediator complex comprises 26 subunits with a molecular weight of approximately 1.4 MDa and is organized into head, middle, tail, and kinase modules [4,5].

Among the Mediator subunits, *MED19* has recently attracted attention because of its emerging role in transcriptional regulation and cancer progression [6]. *MED19* was initially identified in *S. cerevisiae* during a genetic screen for heme-regulated mutants, where it was linked to increased expression of the cytochrome C-7 (*CYC7*) gene [7], and found to cause de-repression of heat-shock genes [8]. Recent studies have demonstrated that *MED19* is frequently overexpressed in multiple tumor types, where it supports cell proliferation, survival, and metastatic potential [9,10,11,12,13,14,15,16,17,18]. These findings suggest that *MED19* functions as a transcriptional co-regulator that supports oncogenic transcriptional programs. However, the downstream transcriptional networks regulated by *MED19* and the extent to which *MED19* dependency varies across cancer types remain largely unclear. Notably, prior studies have primarily established *MED19*’s role at the level of proliferation and survival phenotypes, without defining the specific downstream transcriptional programs mediating these effects or examining whether *MED19* dependency is uniform or context-restricted across cancer types. An emerging strategy in transcription-targeted cancer therapy involves selectively disrupting disease-relevant functions of individual Mediator subunits rather than globally inhibiting the entire complex. Within this framework, *MED19* has emerged as a particularly compelling candidate because of its role in sustaining proliferative and invasive phenotypes across multiple malignancies [5].

In this study, we screened 20 human cancer cell lines representing diverse tissue origins that were available in our laboratory, as an unbiased initial approach to identify cancer types exhibiting differential dependency on *MED19*, using *MED19*-targeting siRNAs and identified A549 and MCF-7 cells as particularly sensitive to *MED19* KD, which were then selected for detailed downstream mechanistic investigation. Flow cytometric analysis showed that *MED19* depletion induced G1-phase arrest, indicating a critical role in G1/S-phase progression. RNA sequencing following *MED19* KD identified *AREG* and *CKS1B* as candidate downstream effectors in A549 and MCF-7 cells, respectively. Depletion of either gene impaired cell proliferation and recapitulated the G1-phase arrest induced by *MED19* KD, whereas their ectopic expression partially rescued the cell-cycle defects. Collectively, these findings identify *MED19* as a context-dependent regulator of G1/S-phase progression and cancer cell proliferation and highlight *MED19*-associated transcriptional programs as potential therapeutic targets in lung and breast cancers.

## 2. Results

### 2.1. Screening Identifies A549 and MCF7 as MED19-Sensitive Cell Lines

**Figure 1:**
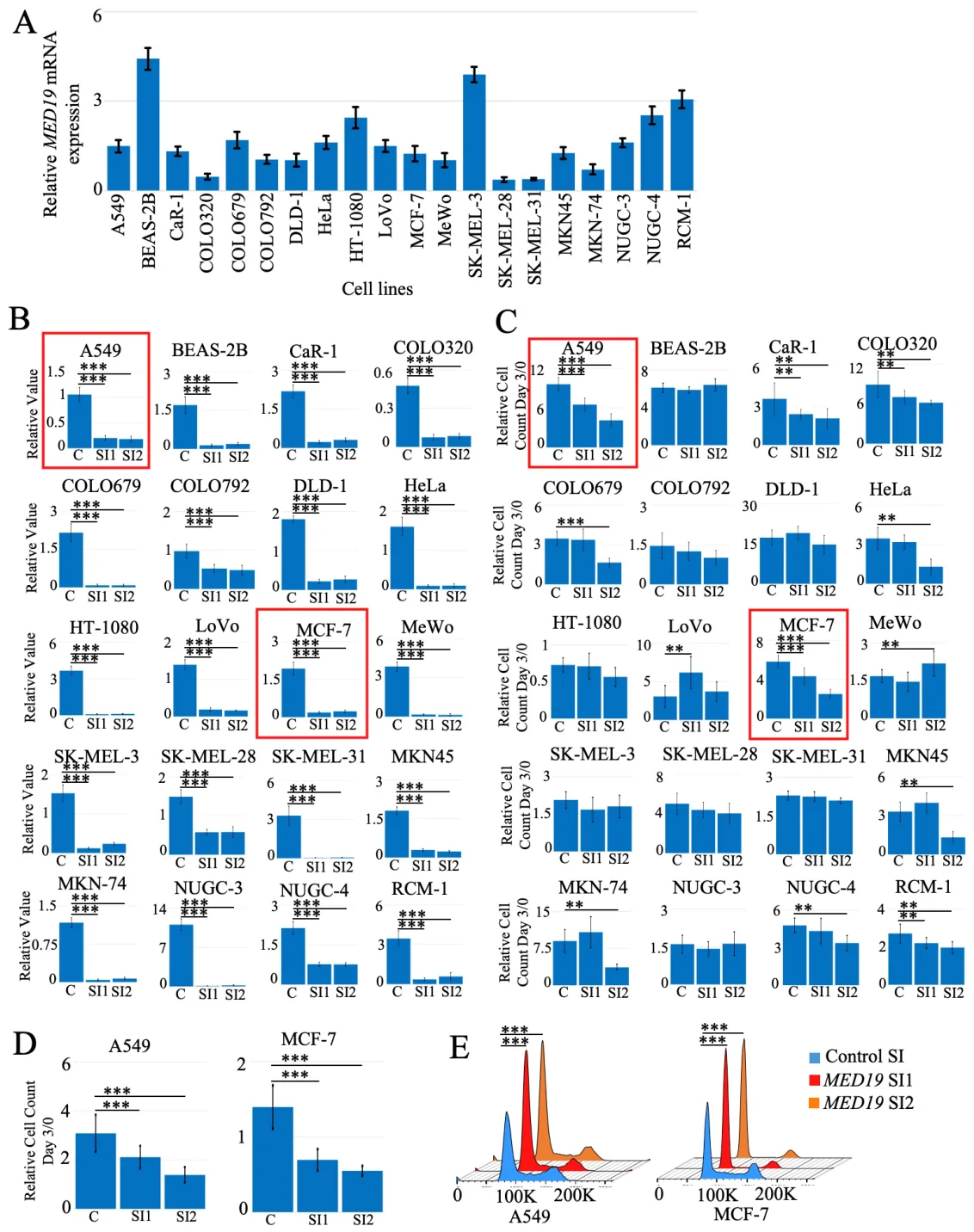
Screening Identifies A549 and MCF7 as *MED19*-Sensitive Cell Lines. (**A**) Basal *MED19* mRNA expression levels across a panel of human cancer cell lines determined by RT-qPCR. Expression levels were normalized to *GAPDH* and presented relative to the reference control. (**B**) Validation of *MED19* KD efficiency following transfection with two independent siRNAs (SI1 and SI2) in the indicated cell lines. *MED19* mRNA expression was analyzed by RT-qPCR 48 h post-transfection and normalized to *GAPDH*. C, control siRNA; SI1, *MED19* siRNA 1; SI2, *MED19* siRNA 2. (**C**) Primary screening of cell viability following *MED19* KD. Relative viable cell numbers were determined by Hoechst 33342 nuclear staining assay 72 h post-transfection. (**D**) Secondary screening of *MED19*-sensitive cell lines using TB exclusion assay. Relative viable cell counts of A549 and MCF-7 cells following *MED19* KD are shown. (**E**) Staggered histogram plots representing altered cell cycle distribution following *MED19* KD in A549 and MCF-7 cells. Data are presented as mean ± SD from at least three independent experiments. Statistical significance was determined relative to the negative control siRNA-transfected cells (*** *p* < 0.001, ** *p* < 0.05).

To determine the cellular dependency on *MED19*, we first examined its expression in 20 cancer cell lines derived from diverse tissue origins and histological subtypes, enabling an unbiased assessment of *MED19* dependency across cancer types prior to focused mechanistic study (Figure 1A). Basal *MED19* expression varied substantially among the cell lines, with several exhibiting markedly elevated *MED19* mRNA levels, suggesting heterogeneity in the regulation of *MED19* transcription.

Next, we performed transient KD of *MED19* using two independent siRNAs in each cell line. RT–qPCR conducted 48 h post-transfection confirmed a reduction in *MED19* transcripts in all tested cell lines (Figure 1B). Although the efficiency of KD varied modestly among cell lines, both siRNAs consistently reduced *MED19* expression compared to negative control. To evaluate the functional impact of *MED19* depletion, we assessed cell viability 72 h post-transfection. Hoechst 33342 nuclear staining revealed a significant reduction in viable cell numbers in a subset of the cancer cell lines, while others showed minimal or no change (Figure 1C). Among the cells lines that were screened, A549, CaR-1, COLO320, MCF-7, and RCM-1 showed significant reductions in cell numbers (*** *p* < 0.0001, ** *p* < 0.05). To validate these findings, a secondary viability screen was conducted using the Trypan Blue (TB) exclusion assay (Figure 1D) for cell lines that exhibited significant decreases in cell viability in the primary screening. Consistent with the Hoechst-based quantification, *MED19* KD only significantly decreased cell viability in A549 and MCF-7 cell lines (*** *p* < 0.0001), whereas CaR-1, COLO320, and RCM-1 cells did not show significant reductions in viability in the secondary assay. This discrepancy likely reflects differences in assay principle: Hoechst 33342 stains nuclei of both live and dead cells and therefore reports relative cell number rather than viability per se, whereas the T B exclusion assay directly measures membrane integrity as a marker of viable cells. CaR-1, COLO320, and RCM-1 are therefore not considered *MED19*-sensitive in this study. To further investigate the mechanism underlying the reduced viability following *MED19* KD, cell-cycle analysis was performed in the *MED19*-sensitive cell lines. Flow cytometric analysis revealed that *MED19* KD using two independent siRNAs significantly increased the proportion of cells in the G1 phase (Figure 1E) (*** *p* < 0.0001). These findings are consistent with previous studies reporting that *MED19* depletion suppresses cancer cell proliferation and induces G0/G1 cell-cycle arrest. In non-small cell lung cancer (NSCLC), KD of endogenous *MED19* inhibited cell proliferation and induced G0/G1 arrest [11]. Similarly, *MED19* suppression reduced cell proliferation and colony-forming ability in breast cancer cells [19] and promoted G1-phase arrest [20]. Comparable effects have also been reported in hepatocellular carcinoma and osteosarcoma models, where *MED19* depletion inhibited cell growth and induced G0/G1 arrest [21,22], suggesting a conserved role for *MED19* in regulating G1/S-phase progression.

Together, these results indicate that although *MED19* is broadly expressed across diverse cancer cell types, only a subset exhibits a strong dependency on *MED19* for proliferation. Moreover, the induction of G1-phase arrest following *MED19* depletion suggests that *MED19* is required for efficient G1/S-phase progression in *MED19*-sensitive cancer cells, highlighting a potential vulnerability that could be therapeutically exploited in selected cancers.

### 2.2. Identification of AREG and CKS1B as Candidate Downstream Effectors of MED19

**Figure 2:**
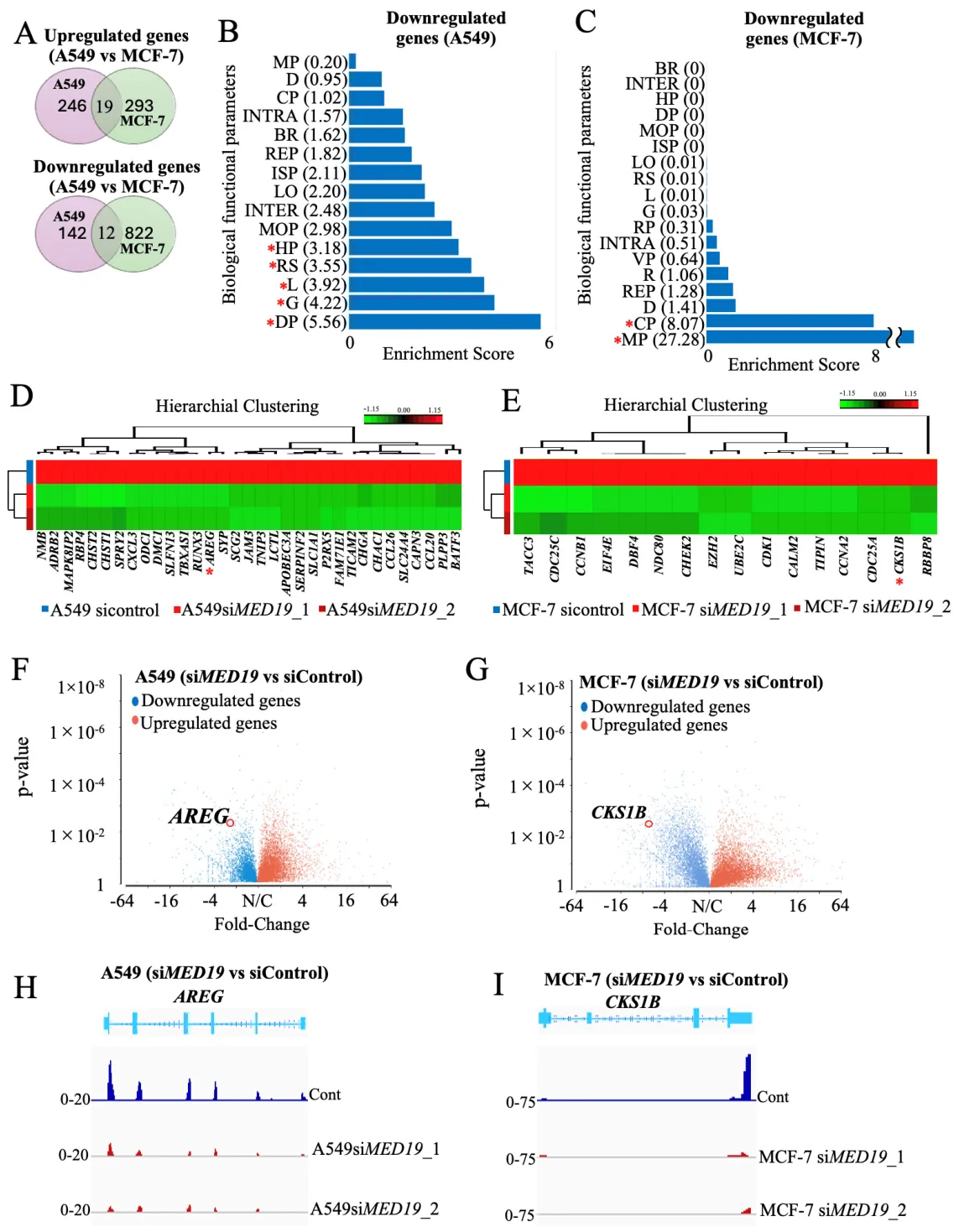
Identification of *AREG* and *CKS1B* as Candidate Downstream Effectors of *MED19.* (**A**) Differential gene expression analysis following *MED19* KD in A549 and MCF-7 cells identified significantly altered transcripts using a cutoff of ≥2-fold change and unadjusted *p* < 0.05 (UAP) relative to negative control cells. (**B**,**C**) GO enrichment analysis of differentially expressed genes (DEGs) performed using Partek Genomics Suite. * Functional categories with enrichment scores > 3 were considered significantly enriched (*p* < 0.05). (**D**,**E**) Hierarchical clustering heatmaps representing expression patterns of DEGs in A549 and MCF-7 cells after *MED19* KD. *AREG* and *CKS1B* are highlighted as candidate downstream targets selected for further investigation. (**F**,**G**) Volcano plots showing genes meeting the ≥2-fold change and UAP < 0.05 threshold in A549 and MCF-7 cells following *MED19* KD, highlighting *AREG* and *CKS1B*. (**H**,**I**) Genome browser chromosome view showing altered transcriptional profiles of *AREG* in A549 cells and *CKS1B* in MCF-7 cells following *MED19* KD. DP: developmental process; G: growth; L: locomotion; RS: response to stimulus; HP: homeostatic process; MOP: multicellular organismal process; INTER: biological process involved in interspecies interaction between organisms; LO: localization; ISP: immune system process; REP: reproductive process; BR: biological regulation; INTRA: biological process involved in intraspecies interaction between organisms; CP: cellular process; D: detoxification; MP: metabolic process; RP: rhythmic process; VP: viral process; R: reproduction.

To identify downstream mediators responsible for the G1-phase arrest induced by *MED19* KD, we performed RNA sequencing (RNA-seq) in A549 and MCF-7 cells following *MED19* depletion. Differential expression analysis revealed substantial transcriptional alterations in both cell lines, and genes exhibiting a ≥2-fold change relative to the negative control were considered differentially expressed (Figure 2A).

To prioritize genes potentially involved in the observed cell-cycle phenotype, Gene Ontology (GO) enrichment analysis was performed using the Partek Genomics Suite. Functional categories with enrichment scores greater than 3 were considered significantly overrepresented (*p* < 0.05). The GO analysis of upregulated genes is presented in Supplementary Figure S1A,B, whereas the enriched categories among downregulated genes are shown in Figure 2B,C. Because *MED19* KD induced G1-phase arrest, we focused on downregulated genes within GO categories associated with cell proliferation, growth, and cell-cycle regulation, reasoning that these genes might mediate the observed phenotype.

In A549 cells, GO analysis identified significant enrichment of genes involved in developmental processes, growth, locomotion, response to stimulus, and homeostatic processes (Figure 2B). To identify genes potentially linked to the G1 arrest phenotype, we generated heatmaps of genes within the response-to-stimulus category. Among these genes, *AREG* was selected for further investigation because it was markedly downregulated following *MED19* KD and displayed a prominent chromosome-view profile (Figure 2D). *AREG* encodes a ligand of epidermal growth factor receptor (*EGFR*) and plays important roles in tissue repair, inflammation, and cellular homeostasis [23]. Upon binding to *EGFR*, *AREG* activates signaling pathways that regulate cell proliferation, survival, and cell-cycle progression. In cancer, *AREG* promotes tumor growth through activation of the PI3K/AKT and MAPK pathways [24,25,26,27,28] and has been implicated in lung cancer progression and resistance to therapy [29,30].

In MCF-7 cells, GO analysis revealed significant enrichment of genes involved in metabolic and cellular processes (Figure 2C). Because the cellular process category contained multiple genes associated with cell-cycle regulation, we further examined genes within the cell-cycle phase transition subcategory. Heatmap analysis identified *CKS1B* as one of the most strongly downregulated genes following *MED19* KD (Figure 2E). *CKS1B* was selected for further investigation because of its established role in G1/S-phase progression and cancer development [31]. *CKS1B* functions as a regulatory component of cyclin-dependent kinase complexes and promotes G1/S transition through SKP2-mediated degradation of the CDK inhibitor p27Kip1 [32,33,34,35]. Elevated *CKS1B* expression has been associated with increased tumor growth, poor prognosis, and therapeutic resistance in multiple cancer types, including breast cancer [31,32,33,34,35].

Volcano plots and chromosome-view analyses further highlighted the marked downregulation of *AREG* and *CKS1B* following *MED19* KD (Figure 2F–I). Together, these analyses identified *AREG* and *CKS1B* as candidate downstream effectors of *MED19* in A549 and MCF-7 cells, respectively. These findings suggest that *MED19* promotes cancer cell proliferation through distinct transcriptional programs that converge on the regulation of G1/S-phase progression.

### 2.3. Functional Validation of AREG and CKS1B as Downstream Effectors of MED19

**Figure 3:**
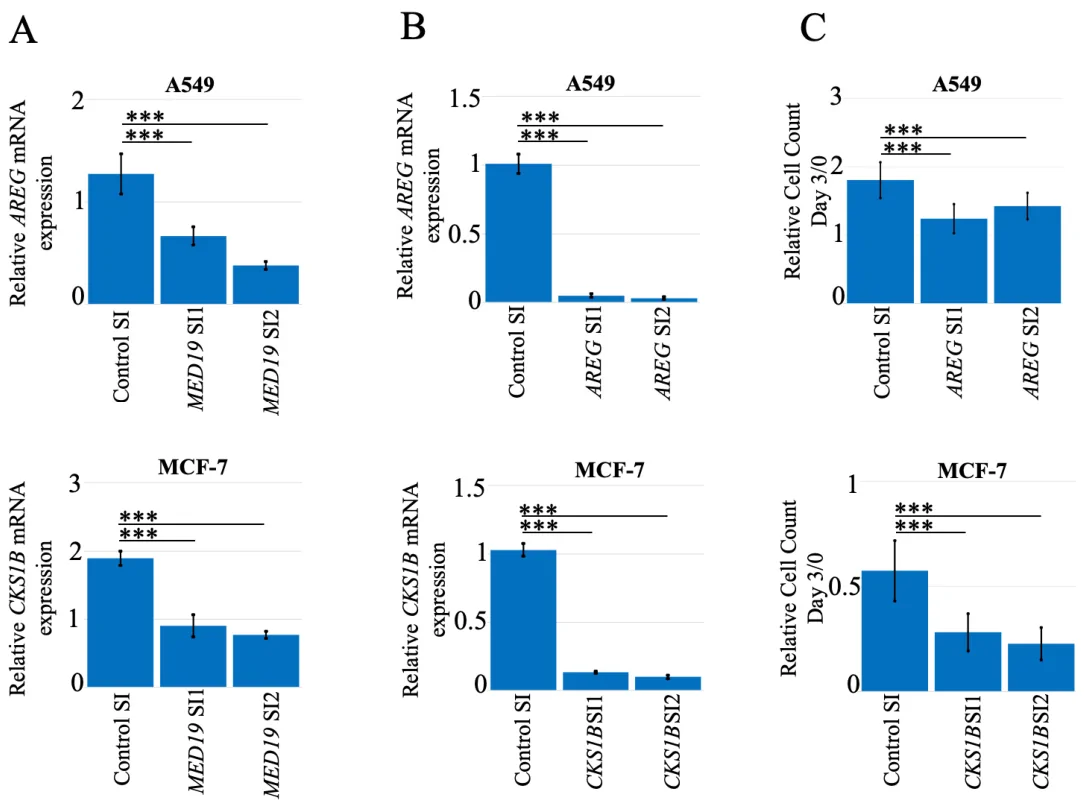
Functional Validation of *AREG* and *CKS1B* as Downstream Effectors of *MED19.* (**A**) RT-qPCR analysis showing the effect of *MED19* KD on *AREG* expression in A549 cells and *CKS1B* expression in MCF-7 cells. *MED19* KD significantly reduced the expression of both target genes. (**B**) Validation of siRNA-mediated KD efficiency for *AREG* in A549 cells and *CKS1B* in MCF-7 cells. Relative mRNA expression levels were analyzed by RT-qPCR 48 h post-transfection and normalized to *GAPDH*. (**C**) Cell viability analysis following *AREG* and *CKS1B* KD using the TB exclusion assay. KD of *AREG* in A549 cells and *CKS1B* in MCF-7 cells significantly reduced viable cell numbers compared with the negative control. Data are presented as mean ± SD from at least three independent experiments. Statistical significance was determined relative to the negative control siRNA-transfected cells (*** *p* < 0.001).

To investigate whether *AREG* and *CKS1B* contribute to the growth-promoting function of *MED19*, we performed siRNA-mediated KD experiments in A549 and MCF-7 cells, respectively. Consistent with the RNA-seq results, *MED19* KD significantly reduced the expression of *AREG* in A549 cells and *CKS1B* in MCF-7 cells, as determined by RT-qPCR (Figure 3A) (*** *p* < 0.0001), further supporting the notion that these genes are regulated by *MED19*.

To directly assess the functional significance of *AREG* and *CKS1B*, each gene was transiently depleted using independent siRNAs. RT-qPCR analysis confirmed efficient KD of *AREG* in A549 cells and *CKS1B* in MCF-7 cells (Figure 3B) (*** *p* < 0.0001). Cell viability was subsequently evaluated using the TB exclusion assay. KD of *AREG* in A549 cells and *CKS1B* in MCF-7 cells resulted in a significant reduction in viable cell numbers compared with the negative control (Figure 3C) (*** *p* < 0.0001), indicating that both genes are required for optimal cancer cell growth.

These findings are consistent with previous studies demonstrating important roles for *AREG* and *CKS1B* in tumor progression. *AREG*, an *EGFR* ligand, promotes cancer cell proliferation and survival through activation of EGFR-dependent signaling pathways, including PI3K/AKT and MAPK cascades. In NSCLC, *AREG* depletion suppresses cell proliferation and colony formation and enhances sensitivity to *EGFR*-targeted therapies [36,37,38]. Likewise, *CKS1B* is a key regulator of cell-cycle progression that facilitates G1/S-phase transition through modulation of cyclin-dependent kinase activity and SKP2-mediated degradation of p27Kip1. Elevated *CKS1B* expression has been associated with enhanced proliferation and aggressive tumor behavior in breast cancer and other malignancies, whereas *CKS1B* depletion impairs tumor cell growth [31,33].

Together, these results demonstrate that *AREG* and *CKS1B* contribute substantially to the proliferative capacity of A549 and MCF-7 cells, respectively. Combined with the RNA-seq analysis, these findings support a model in which *MED19* promotes cancer cell growth through distinct downstream transcriptional programs involving *AREG* in lung cancer cells and *CKS1B* in breast cancer cells.

### 2.4. AREG and CKS1B KD Partially Recapitulate the Cell-Cycle Phenotype Induced by MED19 Depletion

Because *MED19* KD induced G1-phase arrest in both A549 and MCF-7 cells (Figure 1E), we next examined whether depletion of the candidate downstream effectors *AREG* and *CKS1B* could recapitulate this phenotype. Cell-cycle distribution was therefore analyzed following *AREG* KD in A549 cells and *CKS1B* KD in MCF-7 cells.

**Figure 4:**
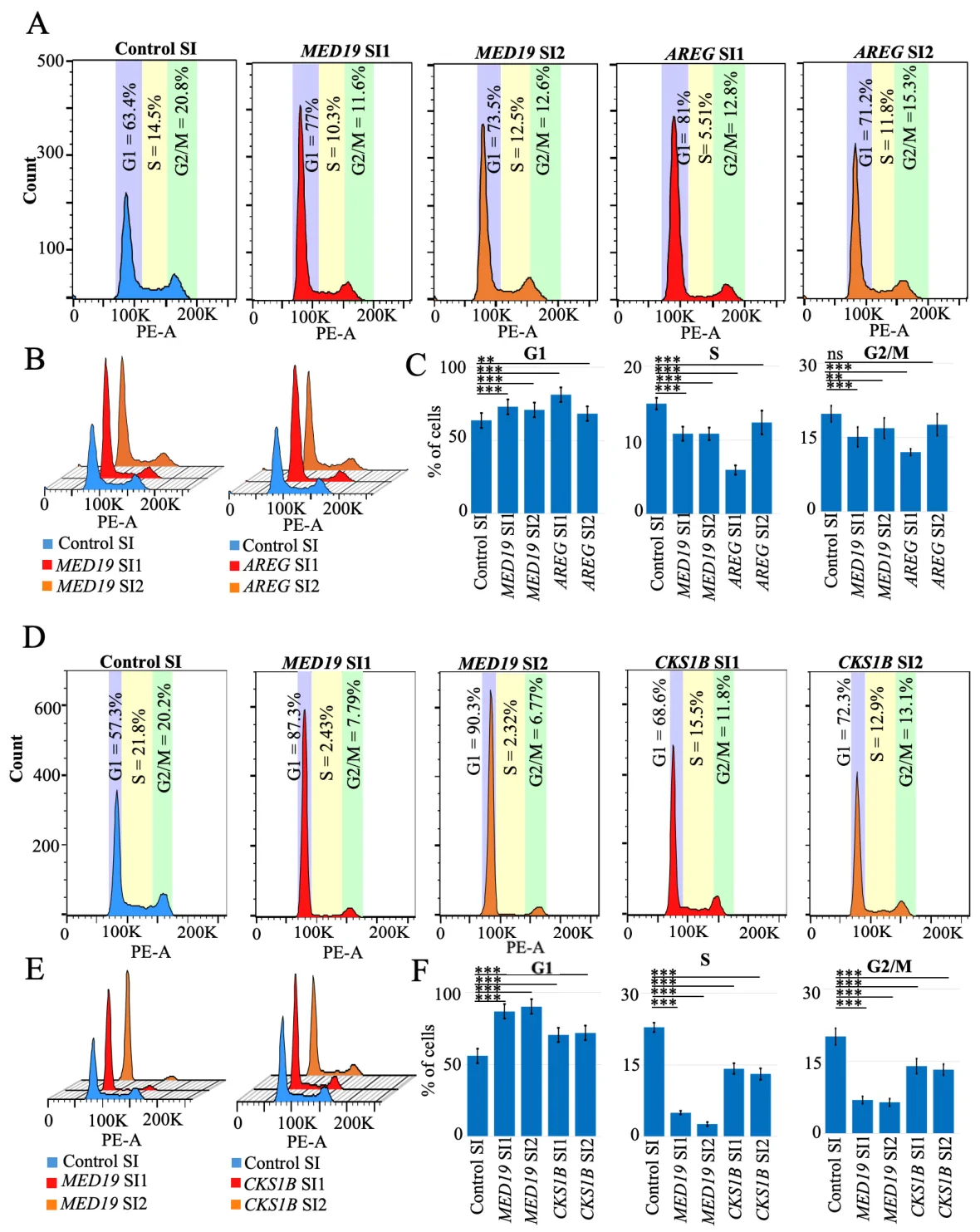
*AREG* and *CKS1B* KD Partially Recapitulate the Cell-Cycle Phenotype Induced by *MED19* Depletion. (**A**,**D**) Flow cytometric analysis of cell cycle distribution following *MED19* and *AREG* KD in A549 cells, and *MED19* and *CKS1B* KD in MCF-7 cells. (**B**,**E**) Staggered histogram plots illustrating altered cell cycle distribution following *MED19* and *AREG* depletion in A549 cells, and *MED19* and *CKS1B* depletion in MCF-7 cells compared with control cells. (**C**,**F**) Quantitative bar plot analysis showing the percentage of cells in G1, S, and G2/M phases following *MED19*, *AREG*, and *CKS1B* KD. (*n* = 5). Data are presented as mean ± SD. Statistical significance was determined relative to the negative control siRNA-transfected cells (*** *p* < 0.001, ** *p* < 0.05; ns, not significant).

In A549 cells, AREG KD resulted in a marked accumulation of cells in the G1 phase, consistent with induction of G1 arrest (Figure 4A). Staggered histogram plots demonstrated a clear shift in cell-cycle distribution following either *MED19* or *AREG* depletion compared with the negative control (Figure 4B). Quantitative analysis further revealed a significant increase in the G1 population accompanied by corresponding decreases in the S- and G2/M-phase populations (Figure 4C) (*** *p* < 0.0001, ** *p* < 0.05). These findings indicate that *AREG* contributes to the maintenance of G1/S-phase progression in A549 cells and suggest that reduced *AREG* expression may account, at least in part, for the G1 arrest induced by *MED19* KD.

Similarly, depletion of *CKS1B* in MCF-7 cells significantly increased the proportion of cells in the G1 phase and reduced the proportion of cells in S phase, demonstrating impaired G1/S-phase progression (Figure 4D). The altered cell-cycle distribution was readily apparent in the staggered histogram plots comparing control, *MED19* KD, and *CKS1B* KD cells (Figure 4E). Quantitative analysis confirmed significant changes in cell-cycle distribution following both *MED19* and *CKS1B* depletion (Figure 4F) (*** *p* < 0.0001, ** *p* < 0.05). Although *CKS1B* KD induced a significant G1 arrest phenotype, the magnitude of the effect was less pronounced than that observed following *MED19* KD. This observation is consistent with the broader role of *MED19* as a transcriptional regulator and suggests that *MED19* likely controls additional genes and pathways that contribute to cell-cycle progression beyond *CKS1B* alone.

The observed phenotypes are consistent with previous reports implicating *AREG* and *CKS1B* in cell-cycle regulation. *AREG* silencing has been shown to impair cancer cell proliferation and induce G1 arrest in colorectal cancer cells, while suppression of *AREG*-dependent signaling can also disrupt G2/M progression through reduced expression of Fork head box M1 (*FOXM1*) target genes in other cancer models [39,40]. Likewise, CKS1 family proteins have long been recognized as regulators of cell-cycle progression. In *Saccharomyces cerevisiae*, Cks1 functions during both G1 and G2 phases of the cell cycle [41]. In mammalian cancer cells, *CKS1B* promotes G1/S-phase progression through SKP2-mediated degradation of the cyclin-dependent kinase inhibitor p27Kip1 [33], whereas depletion of *CKS1B* has also been reported to impair G2/M transition in pancreatic cancer cells [42], Together, these studies suggest that the cell-cycle functions of *AREG* and *CKS1B* may vary depending on cellular context and tumor type.

Collectively, our results demonstrate that depletion of *AREG* or *CKS1B* partially phenocopies the G1 arrest induced by *MED19* KD, supporting their roles as downstream mediators of *MED19*-dependent cell-cycle progression in A549 and MCF-7 cells, respectively. These findings further suggest that *MED19* promotes cancer cell proliferation through distinct transcriptional programs that converge on the regulation of G1/S-phase transition.

### 2.5. Overexpression of AREG and CKS1B Partially Rescue the Cell-Cycle Defects Caused by MED19 Depletion

To determine whether *AREG* and *CKS1B* mediate the cell-cycle regulatory function of *MED19*, we performed rescue experiments in A549 and MCF-7 cells. Cells were transfected with *AREG* or *CKS1B* expression plasmids followed by *MED19* KD, and cell-cycle distribution was subsequently analyzed.

**Figure 5:**
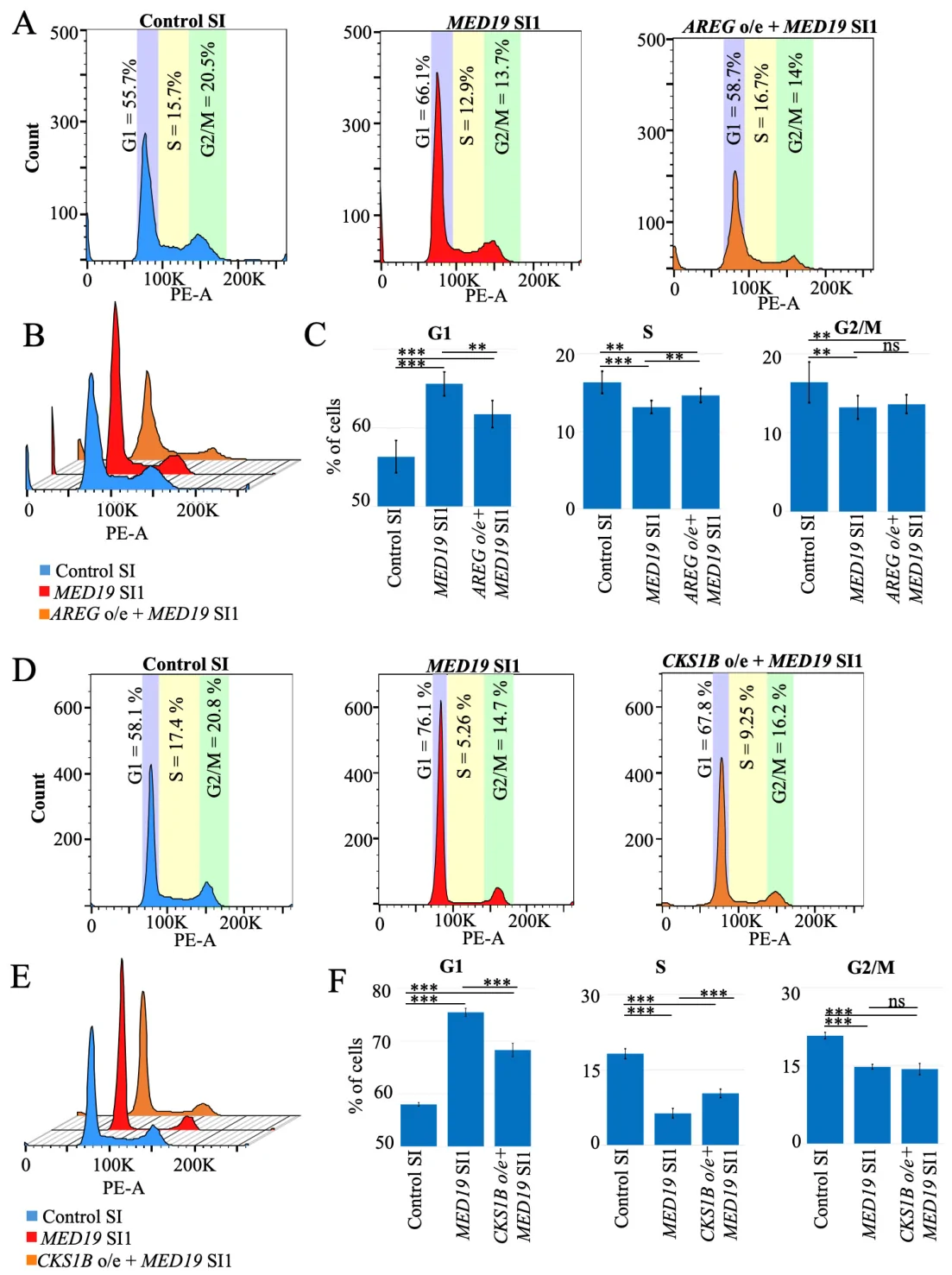
Overexpression of *AREG* and *CKS1B* Partially Rescue the Cell-Cycle Defects Caused by *MED19* Depletion. (**A**,**D**) Flow cytometric analysis of cell cycle distribution following rescue experiments in A549 and MCF-7 cells. Plasmid-mediated overexpression (o/e) of *AREG* in A549 cells and *CKS1B* in MCF-7 cells partially rescued the *MED19*-induced G1 arrest and restored cell cycle progression. (**B**,**E**) Staggered histogram plots illustrating the shift in cell cycle distribution following rescue compared with *MED19* KD alone in A549 and MCF-7 cells. (**C**,**F**) Quantitative bar plot analysis representing the percentage of cells in G1, S, and G2/M phases following rescue experiments. Data are presented as mean ± SD from at least three independent experiments. Statistical significance was determined relative to the negative control siRNA-transfected cells (*** *p* < 0.001, ** *p* < 0.05; ns, not significant).

Consistent with our previous observations, *MED19* KD induced a marked accumulation of cells in the G1 phase, indicative of impaired G1/S-phase progression. Notably, ectopic expression of *AREG* in A549 cells significantly attenuated the G1 arrest phenotype caused by *MED19* depletion and partially restored normal cell-cycle progression (Figure 5A–C). Similarly, overexpression of *CKS1B* in MCF-7 cells significantly rescued the cell-cycle defects induced by *MED19* KD, resulting in a reduction in the G1 population and a corresponding increase in S-phase cells (Figure 5D–F). These rescue effects were statistically significant (*** *p* < 0.0001, ** *p* < 0.05).

The altered cell-cycle distributions were readily apparent in the staggered histogram plots (Figure 5B,E), whereas quantitative analysis confirmed partial restoration of S- and G2/M-phase populations following *AREG* or *CKS1B* overexpression in *MED19*-depleted cells (Figure 5C,F). Importantly, neither *AREG* nor *CKS1B* completely reversed the effects of *MED19* KD, suggesting that additional *MED19*-regulated genes and pathways contribute to cell-cycle control.

To determine whether *AREG* and *CKS1B* overexpression also rescue the proliferation defect associated with *MED19* depletion, we measured relative cell counts (Day 3/Day 0) in A549 and MCF-7 cells; the results are presented in Supplementary Figure S1C,D, respectively. *MED19* KD significantly reduced cell counts relative to control siRNA-transfected cells in both cell lines. Ectopic expression of *AREG* with *MED19* KD in A549 cells significantly increased cell counts relative to *MED19* KD alone, and ectopic expression of *CKS1B* with *MED19* KD in MCF-7 cells similarly increased cell counts relative to *MED19* KD alone; in both cases, there was a partial restoration of proliferation. These findings indicate that *AREG* and *CKS1B* overexpression partially rescue not only the cell-cycle arrest but also the associated proliferative deficit induced by *MED19* KD, reinforcing their functional role as downstream effectors of *MED19* in each respective cell line (*** *p* < 0.001, ** *p* < 0.05).

Consistent with the RT-qPCR data, immunoblotting confirmed these changes at the protein level (Supplementary Figure S1E,F). *MED19* siRNA reduced *MED19* protein in both cell lines, accompanied by reduced *AREG* protein in A549 cells and reduced *CKS1B* protein in MCF-7 cells. In the rescue setting, ectopic expression of *AREG* or *CKS1B* partially restored the respective protein despite persistent *MED19* depletion. These data indicate that the regulatory relationships observed at the mRNA level are maintained at the protein level.

Together, these findings provide functional evidence that *AREG* and *CKS1B* act downstream of *MED19* and contribute to *MED19*-dependent G1/S-phase progression in A549 and MCF-7 cells, respectively. The partial rescue observed in both models further suggests that *MED19* promotes cancer cell proliferation through multiple transcriptional programs that converge on cell-cycle regulation.

## 3. Discussion

Previous studies have established *MED19* as a positive regulator of cancer cell proliferation and tumor progression in multiple malignancies, including NSCLC, breast cancer, hepatocellular carcinoma, and osteosarcoma [11,22,43,44,45]. In these studies, *MED19* depletion consistently impaired cell growth and frequently induced G0/G1 arrest. While these observations implicated *MED19* in cell-cycle regulation, the downstream transcriptional mechanisms responsible for these effects have remained largely unclear.

The present study extends these findings by identifying *AREG* and *CKS1B* as candidate downstream mediators of *MED19*-dependent G1/S-phase progression in lung and breast cancer cells, respectively. Through an unbiased screen of 20 cancer cell lines, we found that sensitivity to *MED19* depletion was restricted to a subset of cell lines, with A549 and MCF-7 cells subsequently selected for mechanistic investigation. In these models, *AREG* and *CKS1B* were identified as downstream candidates based on RNA-seq and GO analysis, together with existing evidence supporting their roles in proliferation and cell-cycle regulation. Functional KD and rescue experiments further supported their involvement in the G1-arrest phenotype induced by *MED19* depletion. These findings suggest that the effects of *MED19* on proliferation may be dependent on cellular context rather than uniformly conserved across cancer types.

*AREG* and *CKS1B* have both been implicated in cancer progression. *AREG*, an *EGFR* ligand originally identified in phorbol 12-myristate 13-acetate (PMA)-treated MCF-7 cells [46], promotes tumor growth and survival through activation of *EGFR* signaling pathways. Elevated *AREG* expression has been associated with poor prognosis and resistance to *EGFR*-targeted therapies in NSCLC [47,48,49,50]. Similarly, *CKS1B* is a well-established regulator of cell-cycle progression and has recently been identified as a potential therapeutic target in breast and colorectal cancers [51]. Aberrant accumulation of *CKS1B* resulting from dysregulated ubiquitin–proteasome pathways has also been linked to tumor progression [52]. The identification of these molecules as downstream targets of *MED19* provides a potential mechanistic explanation for how *MED19* promotes proliferation in distinct cellular contexts.

Our results indicate that *MED19* contributes to G1/S progression, with *AREG* and *CKS1B* identified as downstream candidates in A549 and MCF-7 cells, respectively, based on GO analysis and literature evidence. However, G1/S progression is controlled by a complex network involving cyclins, CDKs, RB pathway components, and E2F target genes. Thus, additional cell-cycle regulators may contribute to the G1 arrest observed following *MED19* KD. Although these pathways were not comprehensively investigated in the present study, future studies examining canonical G1/S regulatory components will be important to further clarify the molecular mechanism underlying *MED19*-mediated cell-cycle regulation.

Although ectopic expression of *AREG* and *CKS1B* significantly alleviated the G1-arrest phenotype induced by *MED19* depletion, the rescue was incomplete. This partial restoration should be interpreted with caution because rescue experiments involving cell-cycle regulators are inherently challenging. The timing and level of ectopic gene expression may not accurately reproduce endogenous regulation during cell-cycle progression, and variable transfection efficiency may further limit rescue efficiency. Therefore, while our findings support functional roles for *AREG* and *CKS1B* downstream of *MED19*, the incomplete rescue indicates that these genes are unlikely to be the sole mediators of the *MED19* depletion phenotype. The additional *MED19*-regulated genes or pathways identified may also contribute to the observed cell-cycle effects. Accordingly, *AREG* and *CKS1B* should be regarded as major, rather than exclusive, downstream effectors of *MED19* in A549 and MCF-7 cells, respectively.

A limitation of this study is that mechanistic and functional validation was restricted to two cell line models (A549 and MCF-7), which, while identified through an unbiased 20-cell-line screen, represent single representative models of lung and breast cancer, respectively. Further validation in additional lung and breast cancer cell lines, as well as in patient-derived organoids, patient-derived xenograft (PDX) models, and clinical specimens (e.g., by immunohistochemistry), will be necessary to establish the broader biological and translational relevance of *MED19*-*AREG* and *MED19*-*CKS1B* signaling. In addition, focused analysis restricted to a single cancer type may reveal additional or more clinically relevant downstream targets beyond *AREG* and *CKS1B*, which the current cross-cancer screening approach was not designed to capture. An additional limitation is that DEGs were defined using an unadjusted *p*-value rather than a multiple-testing-corrected threshold (e.g., FDR); the resulting DEG list should therefore be regarded as a candidate gene set rather than a stringently corrected transcriptomic dataset. Importantly, the principal findings (identification of *AREG* and *CKS1B* as downstream targets of *MED19*) were independently validated by RT-qPCR and functional knockdown/rescue experiments, supporting the conclusions independently of the RNA-seq statistical cutoff. An additional open question is why certain cell lines in our initial screen showed pronounced sensitivity to *MED19* depletion while others were comparatively resistant. Although this study was not designed to dissect the basis of this differential dependency, plausible contributing factors include differences in baseline *MED19* expression, distinct oncogenic driver backgrounds among the sensitive versus resistant lines, and potential functional compensation by other Mediator subunits or transcriptional co-activators in resistant lines. Correlating baseline *MED19* expression levels with KD sensitivity across the full cell line panel represents a straightforward and informative direction for future investigation.

Although the present data cannot formally distinguish whether downregulation of *AREG* and *CKS1B* directly contributes to G1 arrest or is partly a consequence of *MED19*-depletion-induced cell-cycle changes, independent knockdown of *AREG* or *CKS1B* was sufficient to induce G1 arrest, while their overexpression partially rescued the phenotype induced by *MED19* KD. These findings support a functional contribution of *AREG* and *CKS1B* to the cell-cycle effects associated with *MED19* depletion, rather than simply representing passive markers of cell-cycle arrest. Nevertheless, the incomplete rescue indicates that additional *MED19*-regulated factors are likely involved.

In conclusion, our study provides new insight into the transcriptional programs regulated by *MED19* and identifies *AREG* and *CKS1B* as downstream mediators of *MED19*-dependent proliferation in lung and breast cancer cells, respectively. These findings advance our understanding of *MED19* function and suggest that targeting *MED19*-associated transcriptional networks may represent a therapeutic strategy in selected cancers. These findings position *MED19* not as a general driver of proliferation, but as a factor operating through distinct, cancer-type-specific transcriptional programs, supporting a more targeted approach to *MED19*-directed therapeutic strategies.

### Perspectives

The present study identifies *AREG* and *CKS1B* as downstream mediators of *MED19*-dependent cell proliferation and G1/S-phase progression in lung and breast cancer cells, respectively. Although our findings establish functional links between *MED19* and these downstream effectors, the molecular mechanisms through which *MED19* regulates their expression remains to be determined. Given the central role of the Mediator complex in coordinating transcription factor-dependent gene expression, future studies examining *MED19*-associated chromatin occupancy and transcriptional regulatory networks may provide further insight into the context-specific mechanisms underlying *MED19*-driven oncogenic transcription. In addition, in vivo validation will be important to determine the therapeutic relevance of the *MED19*–*AREG* and *MED19*–*CKS1B* regulatory axes in tumor progression.

Because *MED19* is a Mediator subunit and is not expected to bind DNA directly, its effect on *AREG* and *CKS1B* is more likely exerted through sequence-specific transcription factors and RNA Pol II at these loci. Future work should therefore prioritize *MED19* and RNA Pol II ChIP-seq or ChIP-qPCR at the *AREG* and *CKS1B* promoters and associated regulatory regions to test whether *MED19* is recruited there. To establish translational relevance, the findings should be extended to additional lung and breast cancer cell lines, patient-derived organoids, PDX models, and immunohistochemical analysis of clinical tumor specimens. Single-cancer-type studies may also uncover additional or more clinically relevant downstream targets that our cross-cancer screening approach was not designed to detect. Finally, integrating publicly available clinical and genomic datasets would allow *MED19*, *AREG* and *CKS1B* expression, their association with patient prognosis and treatment response, and potential therapeutic vulnerabilities to be evaluated systematically across cancer types. This could help identify the contexts in which targeting the *MED19*–*AREG* and *MED19*–*CKS1B* axes is the most applicable, and which patient populations might benefit.

## 4. Material and Methods

### 4.1. Cell Culture

All 20 cell lines listed in Table S1 were cultured in a humidified chamber (37 °C, 5% CO_2_ atmosphere) (Thermo Forma, Marietta, OH, USA) with Roswell Park Memorial Institute (RPMI) 1640, supplemented with 10 μg/mL gentamicin and 10% fetal calf serum (Life Technologies, Carlsbad, CA, USA). All cell lines used in this study were originally provided by Dr. Tomonori Hayashi (Radiation Effects Research Foundation, Hiroshima, Japan) and were subsequently deposited by Dr. Hayashi in the Japanese Collection of Research Bioresources (JCRB) Cell Bank.

### 4.2. siRNA Transfection

The cells were seeded into 6-well plate at a density of 8 × 10^4^ cells/well for RNA isolation and cell cycle analysis (PI staining). For cell count analysis using Hoechst 33342 and Trypan Blue stains, the cells were seeded at a density of 0.8 × 10^4^ cells/mL and 0.8 × 10^5^ cells/mL into 24-well plates, respectively. The cells were transfected with negative control, *AREG*, *CKS1B*, and *MED19* siRNAs. The sequences of the target siRNAs are given in Table S2.

### 4.3. RNA Extraction and RT-qPCR

After 48hrs of transfection, the total RNA was extracted using the ISOGEN II reagent (NIPPON GENE, Tokyo, Japan). cDNA was synthesized using 0.5 μg of total RNA as a template with oligo (dT) primers (Life Technologies, CA), random hexamers (Takara Bio, Shiga, Japan), and M-MuLV reverse transcriptase (New England Biolabs, Ipswich, MA, USA). RT-qPCR was performed with an ABI PRISM 7900HT (Applied Biosystems, Foster City, CA, USA) with SYBR green as a reporter, cDNA as a template, and gene-specific primers. The target gene expression levels were standardized to *GAPDH* expression levels. All the primers designed for the experiment are listed in Table S3.

### 4.4. Cell Viability (Primary Screening Using Hoechst 33342)

Post transfection, the cells were photographed from day 0 (siRNA transfection) today 3 (72 h post transfection). The cells were stained with Hoechst 33342 for 5 min at 37 °C and photographed. From each well, four images were collected on days 0 and 3. The cells were counted within a 1-mm^2^ area for each image, the day 3 cell counts were normalized to day 0 cell counts and the relative count level was calculated.

### 4.5. Cell Viability (Secondary Screening Using Trypan Blue (TB) Exclusion Dye)

Post transfection, the cells were photographed from day 0 (siRNA transfection) today 3. On day 3, the cells were washed with PBS, trypsinized and pelletized. The cell pellet was dissolved in PBS and 8 μL of sample was mixed with 8 μL of TB (diluted 1:3 in 1× PBS prior to use) and counted using a hemocytometer (ERMA, Tokyo, Japan). Different seeding densities were used for the two assays because the Trypan Blue assay requires cell harvesting and hemocytometer-based counting; preliminary optimization showed that the lower seeding density used for the Hoechst assay yielded insufficient cell numbers for reliable Trypan Blue quantification. A higher seeding density was therefore used for the Trypan Blue assay to ensure accurate, reproducible counts.

### 4.6. RNA-Seq

RNA-seq was carried out using the Illumina MiSeq platform (Illumina, San Diego, CA, USA) according to the manufacturer’s protocol. Total RNA was extracted from A549 and MCF-7 cells transfected with *MED19* siRNAs as previously described [53]. All the RNA-seq data can be found online in the NCBI GEO database (submission GSE334314). Sequencing data were analyzed using the Partek Genomics Suite. Differential gene expression analysis was performed by comparing *MED19* siRNA-treated cells with negative control cells. DEGs were defined as those showing an absolute fold change ≥2 and an unadjusted *p*-value (UAP) < 0.05. GO enrichment analysis was subsequently performed using the Partek Genomics Suite. Functional categories with enrichment scores > 3 were considered significantly enriched (*p* < 0.05).

### 4.7. Plasmid Overexpression

For the *AREG* and *CKS1B* overexpression experiments, the coding sequence (CDS) regions of the *AREG* (NM_001657.4, NCBI) and *CKS1B* (NM_001826.3, NCBI) genes were amplified and cloned into the multiple cloning site (MCS) of the pcDNA3.1(+) vector between the restriction enzyme sites KpnI (GGTACC) and XhoI (CTCGAG), and NotI (GCGGCCGCA) and XhoI (CTCGAG), respectively, thereby generating the overexpression vectors pcDNA3.1-*AREG* and pcDNA3.1-*CKS1B*. For rescue experiments, cells were first transfected with the respective overexpression plasmids for 24 h, followed by siRNA-mediated KD of *MED19* for an additional 48 h. Subsequently, cells were harvested and subjected to Fluorescence-Activated Cell Sorting (FACS) analysis.

### 4.8. PI Staining (Cell Cycle Analysis)

The cells were seeded into 6-well plate at a density of 8 × 10^4^ cells/well. After 48 h, the cells were stained with propidium iodide (PI) (Wako) according to the manufacturer’s instructions and analysed using a FACS Canto II flow cytometer (BD Biosciences, San Jose, CA, USA). The results were analyzed using Flow Jo software version 10.10 (BD Biosciences or Tree Star, Ashland, OR, USA).

### 4.9. Western Blotting

Cells were seeded into 6-well plates at a density of 8 × 10^4^ cells/well. For protein-level validation of siRNA-mediated knockdown, cells were transfected with the respective siRNAs and harvested 48 h after transfection. Cell lysates were prepared using sodium dodecyl sulfate (SDS) sample buffer, separated by 15% SDS-PAGE, and transferred to nitrocellulose membranes (Bio-Rad, Hercules, CA, USA). The membranes were blocked with 3% skimmed milk in TBST (Tris-buffered saline containing 0.05% Tween-20) and incubated overnight at 4 °C with the following primary antibodies: rabbit anti- Mediator complex subunit 19 (*MED19)* (Thermo Fisher Scientific, Waltham, MA, USA, Cat. No. PA5-78656), mouse anti-amphiregulin (*AREG*) (Thermo Fisher Scientific, Cat. No. PA5-102501), rabbit anti- Cyclin-Dependent Kinase Regulatory Subunit 1B (*CKS1B*) (Thermo Fisher Scientific, Cat. No. 37-0200), and anti-core histone (CH) as a loading control. After washing with TBST, the membranes were incubated with HRP-conjugated anti-rabbit or anti-mouse IgG secondary antibodies for 1 h at room temperature. Protein bands were detected using an enhanced chemiluminescence (ECL) substrate and visualized using a chemiluminescence imaging system. Band intensities were quantified using ImageJ software (version 1.54g; National Institutes of Health, Bethesda, MD, USA) and normalized to the corresponding CH loading-control signal. Band intensities were quantified using ImageJ software (National Institutes of Health, Bethesda, MD, USA) and normalized to the corresponding CH loading-control signal.

### 4.10. Statistical Analysis

Data are shown as mean ± SD. Levels of significance for comparison between samples were determined by one-way analysis of variance (ANOVA), followed by Dunnett’s test for the results shown in Figure 1, Figure 3 and Figure 4. Levels of significance for comparison between samples were determined by ANOVA and Tukey’s post hoc test for the results shown in Figure 5 and Supplementary Figure S1. *p*-values of <0.05 were considered statistically significant. All the statistical analyses were performed using JMP Student Edition version 18 software (SAS Institute, Cary, NC, USA). All experiments were conducted with three independent replicates (n = 3) to ensure reproducibility. *** *p* < 0.0001, ** *p* < 0.05.

## Data Availability

The data supporting this study’s findings are available in the methods and Supplementary Materials of this article or in GEO. All the RNA-seq data can be found online in the NCBI GEO database (submission GSE334314).

## Supplementary Materials

The following supporting information can be downloaded at: https://www.mdpi.com/article/10.3390/ijms272110004/s1.

## Abbreviations

ANOVA: one-way analysis of variance; *AREG*: Amphiregulin; BR: biological regulation; *CKS1B*: Cyclin-Dependent Kinase Regulatory Subunit 1B; CP: cellular process; *CYC7*: cytochrome C-7; CDS: coding sequence; D: detoxification; DEGs: differentially expressed genes; DP: developmental process; *EGFR*: Epidermal Growth Factor Receptor; FACS: Fluorescence-Activated Cell Sorting; *FOXM1*: fork head box M1; GO: Gene Ontology; G: growth; HP: homeostatic process; INTER: biological process involved in interspecies interaction between organisms; INTRA: biological process involved in intraspecies interaction between organisms; ISP: immune system process; KD: knockdown; L: locomotion; LO: localization; MP: metabolic process; *MED19*: Mediator complex subunit 19; MOP: multicellular organismal process; NSCLC: non-small cell lung cancer; PI: propidium iodide; PIC: pre-initiation complex; PMA: phorbol 12-myristate 13-acetate; RS: response to stimulus; RP: rhythmic process; R: reproduction; REP: reproductive process; RNA-seq: RNA sequencing; RNA Pol II: RNA Polymerase II; TFs: transcription factors; TB: Trypan Blue; UAP: Unadjusted *p*-value; VP: viral process.
